# Loop-Mediated Isothermal Amplification Technology: Towards Point of Care Diagnostics

**DOI:** 10.1371/journal.pntd.0001572

**Published:** 2012-06-26

**Authors:** Zablon Kithinji Njiru

**Affiliations:** The University of Queensland, The School of Veterinary Sciences, Gatton Campus, Queensland, Australia; Institute of Tropical Medicine, Belgium

## Introduction

The invention of the loop-mediated isothermal amplification (LAMP) method a decade ago has given new impetus towards development of point of care diagnostic tests based on amplification of pathogen DNA, a technology that has been the precinct of well-developed laboratories. The LAMP technology amplifies DNA with high sensitivity relying on an enzyme with strand displacement activity under isothermal conditions. Additionally, the technology uses four to six specially designed primers recognising six to eight regions of the target DNA sequence, hence a high specificity. The auto-cycling reactions lead to accumulation of a large amount of the target DNA and other reaction by-products, such as magnesium pyrophosphate, that allow rapid detection using varied formats [1], [2]. Over the last 10 years, LAMP has been used widely in the laboratory setting to detect pathogens of medical and veterinary importance, plant parasitic diseases, genetically modified products, and tumour and embryo sex identification, among other uses [3]. However, its application under field conditions has been limited, partly due to the infancy of the technologies associated with LAMP, such as field-based template preparation methods and product detection formats. In this Viewpoint, the author highlights the essential technologies that require development before the LAMP platform can be progressed into a realistic point of care format for resource-poor endemic areas.

## ASSURED Tests

Lack of effective point of care diagnostic tests applicable in resource-poor endemic areas is a critical barrier to effective treatment and control of infectious diseases. Indeed, this paucity is acutely demonstrated in neglected tropical diseases (NTDs), where access to reliable diagnostic testing is severely limited and misdiagnosis commonly occurs. Although the reasons for the failure to prevent and control NTDs in the developing world are complex, a major barrier to effective health care is the lack of access to reliable diagnostic laboratory services [4]. The World Health Organization (WHO) recommends that an ideal diagnostic test suitable for developing countries should be Affordable, Sensitive, Specific, User-friendly (simple to perform in a few steps with minimal training), Robust and rapid (results available in 30 min), Equipment free, and Deliverable to the end user (ASSURED) [5]. So far, only a few rapid diagnostic test (RDT) formats fit this model, albeit with limited sensitivity and specificity [6]. Nucleic acid (DNA) amplification tests targeting pathogen markers have high sensitivity and specificity but generally fail to meet the ASSURED guidelines in terms of affordability, rapidity, and being equipment free [7]. However, with the recent invention and advancement of isothermal technologies [8], development of ASSURED tests based on DNA amplification seems realistic. One such potential method is the LAMP technology, which has salient advantages over most DNA-based amplification tests (Box 1). These characteristics make LAMP strategy a potential ASSURED platform. However, for LAMP technology to demonstrate the performance goals implied in the ASSURED guidelines, four technologies that are applicable with the LAMP test need to be developed. These include template preparation protocols, a lysophilised kit, a reliable power source, and product detection technologies.

**Box 1. The Advantages of LAMP Technology**

1. Less expensive (less instrumentation required to achieve amplification)
2. Rapid (results obtained within 1 hour)
3. Sensitivity (equal to or higher to that of classical PCR targeting the same gene)
4. Robust (can amplify target DNA from partially processed or unprocessed specimen)
5. Specificity (high specificity since four to six primes are used targeting six to eight DNA target regions)
6. Product detection (large amount of dsDNA formed and magnesium pyrophosphate allow visual detection formats)
7. Amplification at isothermal conditions (low heat required, hence water bath and exothermal chemical units are sufficient)

## Template Preparation Protocol

**Figure 1:**
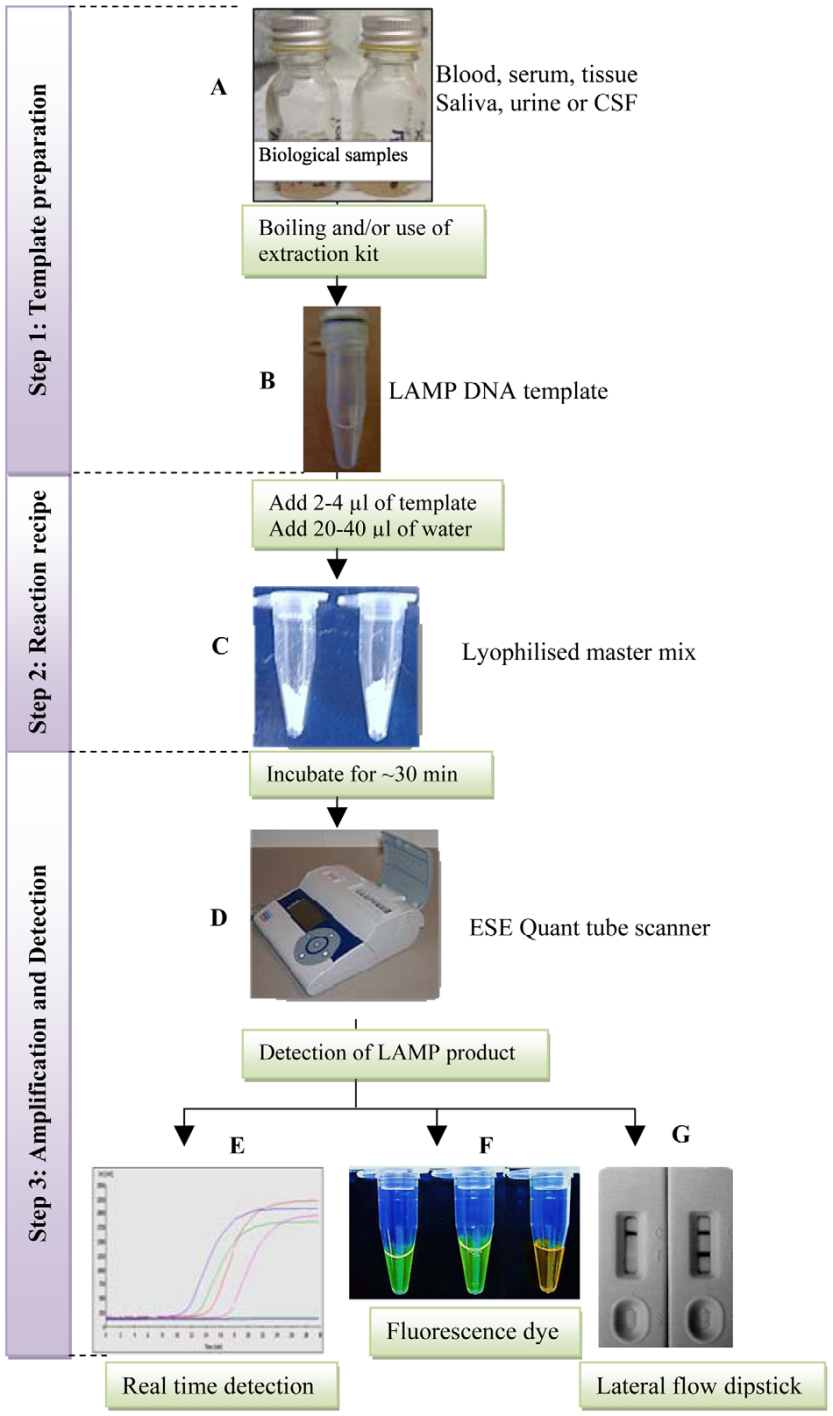
A proposed three-step LAMP method for diagnosis of neglected tropical diseases. Step 1 includes processing of varied specimen (A) through boiling or use of kits to yield a stable and concentrated DNA template (B). In step 2, the lyophilised master mix (C) is reconstituted by the addition of water and the ideal amount of DNA template. In step 3, the amplification and the detection format are combined into a single step to avoid opening the tube (D), hence the results can be acquired in real time (E) through incubation of the reaction with a reporting dye (F) and through the use of a novel LAMP LFD format.

The LAMP method has the advantage of amplifying the target DNA from partially processed and/or non-processed samples [9]. This inherent advantage of LAMP shortens the reaction time and eliminates the need for DNA extraction, a step that is prone to contamination and may result in significant loss of DNA. The preparation of template DNA is the least developed method associated with LAMP technology. For example, the ideal specimens (biological fluids, tissue, swabs, scraping etc.) (Figure 1A) for LAMP reactions are yet to be determined. The direct use of native cerebrospinal fluid, serum, heat-treated blood [10], and addition of detergent [11] have yielded viable DNA templates; however, precise preparation protocols need to be defined and optimised. Nevertheless, these results offer exciting possibilities that definition of a simple field-based template preparation protocol is possible. To improve the performance of LAMP tests, methodologies for specimen collection and processing need to be simple, optimised, and ensure high target yields. In addition, selected buffers should not only stabilise the DNA (reduce degradation in case of storage), but should also enhance amplification whenever possible. An ideal template protocol will thus depend on the sample being tested. For example, whole blood, stool, and tissue specimens may include direct boiling followed by a single buffer-purification step to separate the template from the debris or application of a sample preparation kit that readily removes unwanted biological products followed by the collection and concentration of the template (Figure 1B). Such developments should be approached with the primary aim of reducing cost and potential LAMP inhibitors.

## Lyophilisation of LAMP Recipe

A standard LAMP reaction consists of a large amount of reaction components (reagents), namely the enzyme and its buffer, deoxynucleotide triphosphates, betaine, three sets of primers of varied concentrations, and the detection dye. The researcher/technician is not only faced with the task of acquiring different reagents from varied sources, but the requirement for the cold chain is a major cost during transportation and storage. For example, transporting LAMP reagents from Australia/Europe to Africa requires dry ice as well as expensive flight charges. Furthermore, when the shipment is being sent to multiple recipients, the risk of exposure increases dramatically and is hence a source of varied results. To reduce the test manipulation, Eiken Chemical Co., Japan, developed general LAMP DNA and RNA kits (http://www.eiken.co.jp/en/product/); however, the cold chain is still a requirement. Previous studies indicated that LAMP reagents were stable at temperatures of up to 37°C, raising the possibility of lyophilisation [12], an objective that was recently achieved when Eiken Chemical Co. launched a lyophilised kit for sleeping sickness (http://www.eiken.co.jp/en/news/pdf/20110916-1.pdf). Another experimental lyophilised LAMP kit for animal diseases is under evaluation by the International Atomic Energy Agency (IAEA) and the results are promising (Herman Unger, personal communication). Since different pathogen biomarkers operate under different LAMP conditions [13], development of a general LAMP kit that requires only addition of specific pathogen primers is not feasible. As such, an ideal LAMP kit will be disease specific and possibly consist of a lyophilised master mix (including the primers) and only require re-constitution and sample addition (Figure 1C). Moreover, the kit should be affordable and stable under hot and humid tropical conditions.

## Reliable Power Source

One of the primary barriers to the practicality of LAMP under field conditions is the requirement of electricity to run the test and refrigerate the reagents. However, LAMP reactions are rapid and proceed at a lower temperature (∼60°C) compared to PCR, meaning that less time and heat is required to complete incubation. This is attributable to the use of DNA polymerase with a strand displacement activity. The prevailing conditions in most disease-endemic regions dictate that the source of power for ASSURED tests needs to be reliable and possibly portable within rugged environmental conditions; therefore, electricity-free technologies such as a water incubator heated with wood or charcoal, re-chargeable batteries (including solar based), engineered mechanical devices, and exothermal chemical units are most appropriate. Indeed, such simple to operate prototype devices have been developed based on calcium oxide or quicklime [14] and a battery-operated and portable fluorescence reader (ESEQuant Tube Scanner, Qiagen ESE Gmbh, Stockach, Germany) (Figure 1D). Among these, the ESEQuant Tube Scanner is most advanced and has several advantages in that it offers (i) a single-step amplification and product detection, (ii) results can be monitored in real time using a program installed in a laptop (Figure 1E) and/or through an LCD window, (iii) a re-chargeable battery, and (iv) is simple to operate, small 74 (h)×178 (w)×188 mm, and light weight (1 kg). The downside of the unit is that it runs only eight samples and uses non-specific dye for detection of double-stranded DNA (dsDNA). Nevertheless, the ESEQuant Tube Scanner provides the first major advancement towards “electricity free” technology for LAMP technology. The next major step is rigorous evaluation of the ESEQuant Tube Scanner and possibly its modification to accommodate more samples. Other potential sources of heat include sodium acetate (heat pads), which can be packaged into customised and insulated units. Such a device will require clicking a metal disc to initiate nucleation and crystallization to reach temperatures of ≥54°C for over an hour and sufficient for LAMP reactions using *Bsm* DNA polymerase (unpublished data). Sodium acetate is non-toxic and the heat pads can easily be recharged by boiling in water and reused many times.

## Detection of LAMP Product

Significant progress has been made with modification of the LAMP method, but the product detection technologies have not seen similar advancement. The LAMP reaction produces large amounts of magnesium pyrophosphate (a white precipitate) and dsDNA, which allow visual inspection of results using a turbidimeter and real-time PCR machine, respectively, avoiding post DNA amplification manipulation (Figure 1E and 1F). To achieve a white precipitate, extra Mg^++^ is required, which may compromise test specificity [10], while fluorescence dyes have a major limitation in that they can bind non-specifically to any dsDNA, such as primer-dimers, leading to erroneous result interpretation. Other reported non-specific dyes include calcein and and hydroxy naphthol blue, which are incubated with the reaction, and SYBR Green I, which is added after the reaction, a step that may lead to subsequent contamination once the tubes are opened. A higher test specificity can be achieved by targeting an internal sequence of the amplicon through incorporating fluorescent molecular beacon probe, thus minimising non-specific signal [15], or by using a lateral flow dipstick (LFD) format (Figure 1G) [16], though at a slightly higher cost. In general, the use of dyes is most preferred because they are cheap and most allow definite visual inspection of results based on colour change [17]. However, the need for treatment and/or a decision on case management dictates unequivocal result interpretation. Thus, any LAMP detection format should not compromise specificity for simplicity. An ideal LAMP detection format would thus include a closed amplification and detection unit to limit contamination and use of a dye of acceptable specificity and/or a novel LFD format. For example, it is worth noting that the human African trypanosomiasis case definition requires demonstration of trypanosomes in the body fluid, and that the LAMP test may not be relied upon to make a treatment decision. Nevertheless, the expected high specificity of LAMP may reduce costs associated with serological false positives that card agglutination test for trypanosomiasis (CATT) registers, of which only few end up being genuine HAT cases.

## Future Direction

The way forward for LAMP as a point of care diagnostic platform requires a continuing quest to advance the technology into ASSURED format. A field-based LAMP test may consist of three to four diagnostics steps or fewer, depending on the type of specimen (Figure 1). Achieving such formats require concerted innovative research efforts, an objective that is strained by lack of funding. Noticeable achievement has been made through collaboration between academia and philanthropic organisations. In this context, the Foundation for Innovative New Diagnostic (FIND), Geneva, academia, and Eiken Chemical Co. have progressed the repetitive insertion mobile element (RIME) LAMP for sub-genus *Trypanozoon* (sleeping sickness) into a field-applicable format. Furthermore, the Stop Buruli initiative and the University of Queensland are working towards a field-based Buruli ulcer disease test format. Since considerable progress has been gained in advancement of the LAMP platform, more efforts need to be directed towards development of the associated technologies. This should be followed by rigorous evaluation of test performance to determine feasibility and acceptability under field conditions. It is the completion of these indispensible technologies that will lead to realisation of a LAMP-based point of care test. Since LAMP technology shows much potential as a diagnostic tool for NTDs, sustainable programmes for quality assurance need to be established alongside various technological formats. Further, to ensure a continued production of kits beyond project life, incorporation of a commercialisation plan is advisable.

## Conclusion

Diagnostic testing, and in particular early detection, is critical for most NTDs, as most infected individuals are often asymptomatic but nonetheless sick and infectious. As such, sensitive diagnostic tests are required to guide treatment, minimise drug exposure to non-infected individuals, and interrupt disease transmission. The rapid pace in LAMP technology development and the current support from philanthropic groups and other funding agencies makes it more likely that in the next 5 years we may have specific LAMP tests applicable at the point of care. It is suggested here that the technologies associated with LAMP be considered and developed as part of a LAMP platform, rather than developing them as separate entities. This will mean apportioning the available funding in developing these formats. Considering LAMP's simplicity in operation and high sensitivity, there is potential use in clinical diagnosis and surveillance of infectious diseases. To achieve these levels in resource-poor endemic areas, specimen processing methods, production of lyophilised kits, and a closed amplification and detection system need to be developed. This will facilitate the provision of a same-day testing strategy in even the most remote rural health facilities.
